# Thrombectomy with and without emergent stenting in acute ischemic stroke due to carotid artery dissection

**DOI:** 10.1093/esj/aakaf004

**Published:** 2025-12-28

**Authors:** Lisa Kaindl, Michail Giannakakis, Joshua Mbroh, Alexandra Gomez Exposito, Stefan Krebs, Julian Hotz, Dominika Miksova, Mira Katan, Susanne Wegener, Gian Marco De Marchis, Thomas Gattringer, Hannes Deutschmann, Lukas Mayer-Suess, Jens Fiehler, Ulrike Ernemann, Florian Hennersdorf, Urs Fischer, Zsolt Kulcsar, Pasquale Mordasini, Marios-Nikos Psychogios, Elke Ruth Gizewski, Christian Nolte, Christian Neumann, Julia Ferrari, Tomas Dobrocky, Sven Poli, Marek Sykora

**Affiliations:** Department of Neurology, St. John’s Hospital Vienna, Vienna, Austria; Department of Neurology, University Hospital and University Basel, Basel, Switzerland; Department of Neurology & Stroke, University of Tübingen, Tübingen, Germany; Hertie Institute for Clinical Brain Research, University of Tübingen, Tübingen, Germany; Department of Neurology & Stroke, University of Tübingen, Tübingen, Germany; Hertie Institute for Clinical Brain Research, University of Tübingen, Tübingen, Germany; Department of Neurology, St. John’s Hospital Vienna, Vienna, Austria; Department of Neurology, St. John’s Hospital Vienna, Vienna, Austria; Gesundheit Österreich GmbH, Vienna, Austria; Department of Neurology, University Hospital and University Basel, Basel, Switzerland; Department of Neurology, Universitätsspital Zürich, Zürich, Switzerland; Department of Neurology, University Teaching & Research Hospital, HOCH Cantonal Hospital St. Gallen, St. Gallen, Switzerland; Department of Neurology, Medical University Graz, Graz, Austria; Division of Neuroradiology, Vascular and Interventional Radiology, Department of Radiology, Medical University Graz, Graz, Austria; Division of Neuroradiology, Vascular and Interventional Radiology, Department of Radiology, Medical University Graz, Graz, Austria; Department of Neurology, Medical University Innsbruck, Innsbruck, Austria; Department of Neuroradiology, University Hamburg, Hamburg, Germany; Department of Neuroradiology, University Tübingen, Tübingen, Germany; Department of Neuroradiology, University Hamburg, Hamburg, Germany; Department of Neurology, University Hospital Bern, University of Bern, Bern, Switzerland; Department of Neuroradiology, Clinical Neuroscience Center, University Hospital of Zürich, Zürich, Switzerland; Institute of Diagnostic and Interventional Neuroradiology, Kantonsspital Aarau (KSA), Aarau, Switzerland; Department of Neuroradiology, University Basel, Basel, Switzerland; Department of Radiology, Medical University Innsbruck, Innsbruck, Austria; Department of Neurology with Experimental Neurology, Charité Universitätsmedizin Berlin and Center for Stroke Research (CSB), Berlin, Germany; Department of Radiology, St. John’s Hospital Vienna, Vienna, Austria; Department of Neurology, St. John’s Hospital Vienna, Vienna, Austria; Department of Neuroradiology, University Hospital Bern, Bern, Switzerland; Department of Neurology & Stroke, University of Tübingen, Tübingen, Germany; Hertie Institute for Clinical Brain Research, University of Tübingen, Tübingen, Germany; Department of Neurology, St. John’s Hospital Vienna, Vienna, Austria; Sigmund Freud University Vienna, Vienna, Austria

**Keywords:** thrombectomy, stenting, dissection, stroke, outcome

## Abstract

**Introduction:**

Whether thrombectomy with or without emergent carotid stenting improves outcomes in patients with large vessel occlusion (LVO) stroke due to carotid artery dissection (CAD) is unknown.

**Patients and methods:**

International multicentre observational study. Patients with LVO due to CAD undergoing thrombectomy with emergent stenting were compared to those without emergent stenting. The primary outcome was functional independence (modified Rankin Scale 0-2) at 3 months, secondary outcomes included early neurological improvement (ENI) within 24-48 h, successful recanalisation, symptomatic intracerebral haemorrhage (sICH) and mortality at 3 months. Inverse probability of treatment weighting and multivariable Poisson regression were used to adjust for group imbalances and to estimate the effect size, respectively.

**Results:**

Of 516 patients (mean age 53.8 years, 76% male) undergoing thrombectomy, 167 (32.4%) and 349 (67.6%) were treated with or without emergent carotid stenting, respectively. After robust adjustment, emergent stenting was not associated with functional independence (adjusted risk ratio [aRR] = 1.01; 95% confidence interval [CI], 0.89-1.15) or ENI (aRR = 1.07; 95% CI, 0.95-1.21) but with successful recanalisation (aRR = 1.29; 95% CI, 1.10-1.50) and reduced mortality at 3 months (aRR = 0.39; 95% CI, 0.15-0.99). Risk of sICH was equivalent (aRR = 1.01; 95% CI, 0.95-1.06).

**Conclusion:**

In patients with LVO secondary to CAD, emergent stenting during endovascular procedure appeared safe, increased odds of successful recanalisation and reduced 3-month mortality rates. However, intraprocedural stenting was not associated with better functional outcome.

## Introduction

Carotid artery dissection (CAD) is a major cause of acute ischemic stroke in young and middle-aged adults.^1-3^ Occlusion of a dissected artery is an independent predictor of the unfavourable functional outcome.^4^ Although thrombectomy is an established treatment for acute stroke due to large vessel occlusion (LVO) regardless of aetiology,^5-9^ data on the optimal endovascular management of patients with LVO due to CAD are limited.^10-12^ Dissection presents a technical challenge that may lead to higher complication rates and prolonged procedure times.^13-15^ Evidence supporting carotid artery stenting during thrombectomy is mostly based on cases of atherosclerotic occlusions.^10^^,^^11^^,^^14^^,^^16-18^ However, these results may not be applicable to dissections.^10^^,^^11^^,^^13^^,^^19^ The aim of our study was to evaluate outcomes and safety of thrombectomy with and without emergent stenting in patients with LVO in the anterior circulation due to CAD.

## Patients and methods

### Study design and population

This study was a secondary analysis of the CONCORDIA (Carotid dissectiON thrombeCtOmy veRsus meDIcal treAtment) collaboration study published previously.^20^ Shortly, CONCORDIA was an investigator-driven, international, multicentre, observational study pooling data from 3 nationwide stroke registries in Austria, Germany and Switzerland. The Austrian Stroke Unit Registry is a prospective database collecting data of all patients with stroke treated in 1 of 38 Austrian stroke units. Founded in 2003 and administrated by the Federal Ministry of Health, the registry includes anonymised patients’ data including epidemiologic, demographic, clinical, therapeutical and outcome variables using a web-based interface.^21^ German Stroke Registry-Endovascular Treatment (GSR-ET) is an academic, ongoing, prospective, open-label, multicentre registry comprising patients who underwent thrombectomy at 1 of 25 comprehensive stroke centres in Germany (ClinicalTrials.gov NCT03356392).^22^ Swiss Stroke Registry (SSR) is a national prospective hospital-based registry implemented in the clinical data management system secuTrial. Since 2014, all consecutive stroke patients hospitalised in stroke units and/or comprehensive stroke centres (all certified according to national Swiss Stroke Unit and Stroke Center criteria [https://www.sfcns.ch/Stroke.html], and those of the European Stroke Organisation) must be enrolled in the SSR, which was designed for quality control and research in acute stroke management. The registry is governed by the Swiss Stroke Society.^23^ Data collection, clinical ratings and data entry in all 3 registries are performed by vascular neurologist at the respective stroke unit upon admission, discharge and at 3 months follow-up using standardised definitions of variables and scores.

Eligible patients for the study were required to be 18 years or older, undergoing thrombectomy with or without intraprocedural emergent carotid stenting between 2018 and 2023 for LVO in the anterior circulation associated with CAD. Diagnosis of CAD was based on the respective neuroradiological findings and judgement of the treating neuroradiologist and/or stroke neurologist. The decision whether to use thrombectomy with or without stenting as well as periprocedural medical management was not influenced by study participation but left to the treating physician’s discretion. The rationale for stenting was to restore the flow within the ICA to optimise cerebral reperfusion and to prevent recurrent thromboembolic events. All assessments, including admission National Institutes of Health Stroke Scale (NIHSS), modified Rankin Scale (mRS) at 3 months or recanalisation status post thrombectomy, were performed on-site by experienced neurologists and neuroradiologist.

### Study outcomes

The primary endpoint was functional independence (mRS 0-2) at 3 months. Secondary endpoints included symptomatic intracerebral haemorrhage (sICH) as defined by the European Cooperative Acute Stroke Study (ECASS) III criteria (ie, an ICH associated with an increase of ≥ 4 points on the NIHSS or death where intracranial haemorrhage is the predominant cause of neurological deterioration), recanalisation success (Treatment in Cerebral Ischemia [TICI] Score 2b/3) early neurologic improvement (ENI, decrease of NIHSS ≥ 4 within 24-48 h), early neurologic deterioration (END, increase of NIHSS ≥ 4 within 24-48 h) and mortality at 3 months.

### Standard protocol approvals, registrations and patient consents

Data collection and analyses were conducted in accordance with national rules of approval and supervised by the respective academic review boards. The study protocol was approved by the local ethics committees (AT: EK SFU, Nr. 1025-2024; CH: EK NZ 2023-00796), with patients in the German Stroke Registry having provided informed consent for future analyses (DE: EK LMU 689-15, EK UKT 057/2016BO2). Given the retrospective and anonymised design of this study, written informed consent was not required in Austria and Switzerland.

### Data availability statement

Data that support the findings of this study are available from the corresponding author after national academic boards review upon reasonable request.

### Statistics

Results are presented as median and interquartile range for continuous variables, while categorical variables are summarised by absolute frequencies (*n*) and relative frequencies (%). Patients were categorised according to the presence of intraprocedural emergent stenting. In the univariate analysis, the 2 respective groups were compared using the Mann–Whitney *U*-test and Pearson’s chi-square as appropriate. Inverse probability of treatment weighting (IPTW) was used to adjust for group imbalances as follows: propensities were calculated using a multivariable logistic regression model including factors such as age, sex, pre-stroke mRS, hypertension, diabetes, atrial fibrillation, admission NIHSS, occlusion localisations (M1-segment, M2-segment of the middle cerebral artery, carotid-T, anterior cerebral artery, isolated extracranial carotid artery occlusion, tandem occlusion), intravenous thrombolysis and centre, and transformed into stabilised weights. The extreme weights were handled using truncation. Unweighted and weighted cohorts were compared using standardised mean differences (SMDs) to assess residual bias. Weighted multivariable Poisson regression with a robust variance estimator was then applied to estimate the effect sizes for the primary and secondary outcomes. The model was adjusted for clinically relevant confounders and confounders not achieving SMD < 0.1 after IPTW (age, sex, atrial fibrillation admission NIHSS and intravenous thrombolysis). Subgroup analysis for stroke severity (admission NIHSS ≤ 6 vs > 6), tandem occlusion, isolated extracranial carotid artery occlusion and for intravenous thrombolysis versus no intravenous thrombolysis were performed using stratification and multiplicative interaction terms. Missing data were handled as follows: for nearly all confounders and outcomes the missing values were below 5% and were thus ignored. For TICI scores multiple imputations were used, for mRS at 3 months a sensitivity analysis was calculated. All statistical analysis was performed using IBM SPSS statistical software (version 29) and R software (version 4.3.2).

## Results

### Study population

Between 2018 and 2023, 1023 patients with acute stroke caused by LVO in the anterior circulation secondary to CAD were included into Austrian, German and Swiss Stroke Registries. Of those, 516 received endovascular therapy. 349 (67.6%) were treated with and 167 (32.4%) without intraprocedural emergent extracranial stenting, respectively. Three-month functional outcome was available for 415 (80.4%) patients. Characteristics of those with and without 3-month follow-up did not differ significantly (Supplementary Table S1).

**Figure 1:**
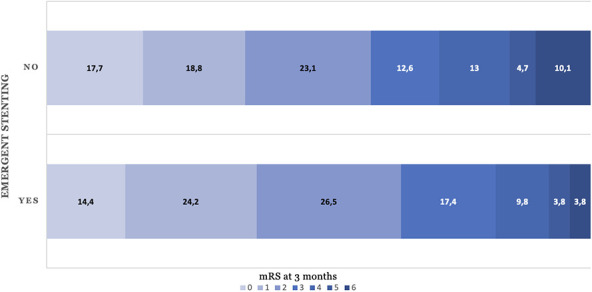
Distribution of functional outcome at 3 months of thrombectomy for large vessel occlusion due to carotid dissection categorised by emergent carotid stenting. Bar graph showing the distribution of modified Rankin Scale (mRS) scores at 3 months after thrombectomy in patients with large vessel occlusion due to carotid artery dissection, stratified by whether emergent carotid stenting was performed.

### Baseline characteristics

A summary of the baseline characteristics of the study population is provided in Table 1. Of 516 included patients, 393 (76%) were male. The mean age was 53.8 years. Except for occlusion site, all assessed baseline factors did not differ statistically significant between the treatment groups. Among patients undergoing endovascular therapy, those with intraprocedural emergent stenting more frequently had M2 occlusions (55 [32.9%] vs 81 [23.2%], *P* = .02), whereas patients without stenting more often presented with M1 occlusions (185 [53%] vs 72 [43.1%], *P* = .04) (Table 1). After IPTW, patient characteristics in the groups with and without emergent stenting were equally distributed (Supplementary Table S2).

**Table 1:** Characteristics of the study population categorised by emergent carotid stenting.

	**No stent = 349**	**Stent = 167**	***P*-value**
Age, mean (range, SD)	53.2 (19-95, 12.6)	55.1 (27-85, 10.4)	.1
Sex, *n* (%) male	258 (73.9)	135 (80.8)	.1
Admission NIHSS, median (range, IQR)	14 (0-40, 10)	13 (0-40, 11)	.4
Pre-stroke mRS 0-1, *n* (%)	330 (94.6)	160 (95.8)	.7
Hypertension, *n* (%)	145 (41.5)	76 (45.5)	.5
Diabetes mellitus, *n* (%)	14 (4)	10 (6)	.4
Atrial fibrillation, *n* (%)	16 (4.6)	7 (4.2)	1
Vessel occlusion localisation			
M1, *n* (%)	185 (53)	72 (43.1)	.04
M2, *n* (%)	81 (23.2)	55 (32.9)	.02
Carotid-T, *n* (%)	78 (22.3)	43 (25.7)	.5
Isolated extracranial ICA, *n* (%)	97 (27.8)	57 (34.1)	.15
ACA, *n* (%)	10 (2.9)	4 (2.4)	1
Tandem, *n* (%)	58 (16.6)	31 (18.6)	.6
Intravenous thrombolysis, *n* (%)	169 (48.1)	78 (46.7)	.78
Door-to-needle time, min; median (range, IQR)	36.5 (4-195, 39.75)	40 (5-975, 43)	.8
Door-to-groin time, min; median (range, IQR)	75 (11-532, 66)	79 (9-680, 56.5)	.25
Duration of EVT, min; median (range, IQR)	72 (14-541, 65.75)	61 (8-420, 85)	.9

Abbreviations: SD = standard deviation; IQR = interquartile range; NIHSS = National Institute of Health Stroke Scale; mRS = modified Rankin Scale, M1 and M2—M1 and M2 segment of the middle cerebral artery, ICA = internal carotid artery, ACA = anterior cerebral artery, EVT = endovascular therapy.

### Primary and secondary outcomes

A total of 86 of 169 (65.2%) and 165 of 349 (59.6%) patients undergoing thrombectomy with and without carotid artery stenting achieved favourable outcome mRS 0-2 at 3 months, respectively. After adjustment, stenting was not associated with favourable functional outcome (aRR = 1.01; 95% confidence interval (CI), 0.89—1.15). Early neurological improvement (ENI) occurred in 94 patients (55.6%) with CAS and in 182 patients (52.1%) without CAS, aRR = 1.07; 95% CI, 0.95-1.21, while early neurological deterioration (END) was observed in 22 patients (35.5%) and 52 patients (38.8%), aRR = 0.91; 95% CI, 0.62-1.34, respectively. Thrombectomy with stenting as compared to no stenting led to significantly higher rates of successful recanalisation: 153 (90.6%) versus 271 (77.6%) patients achieved TICI 2b/3. After adjustment, thrombectomy with stenting was associated with TICI 2b/3 with aRR = 1.29; 95% CI, 1.10-1.50 (Table 2). At 3 months, the mortality rate was lower among patients who underwent intraprocedural stenting (5 [3.8%]) compared to those who did not (28 [10.1%]) with aRR = 0.39; 95% CI, 0.15-0.99. Risk of sICH was equivalent between treatment groups (4.8% vs 4.3%), aRR = 1.01; 95% CI, 0.95-1.06 (Table 2, Figure 1).

**Table 2:** Outcomes of endovascular therapy with and without emergent stenting for LVO due to carotid dissection.

	**No stent** ***n* = 349**	**Stent** ***n* = 167**	**RR (95% CI)**	***P*-value **	**aRR (95% CI)**	***P*-value **
mRS 0-2 at 3 months (*n* = 409)	165 (59.6)	86 (65.1)	1.27 (0.82-1.95)	.33	1.01 (0.89-1.15)	.88
ENI	182 (52.1)	94 (55.6)	1.16 (0.78-1.73)	.48	1.07 (0.95-1.21)	.25
sICH	15 (4.3)	8 (4.8)	1.12 (0.46-2.69)	.82	1.01 (0.95-1.06)	.88
TICI 2b/3	271 (77.6)	153 (90.6)	2.83 (1.12-7.17)	.03	1.29 (1.10-1.50)	<.001
Mortality at 3 months	28 (10.1)	5 (3.8)	0.38 (0.15-0.95)	.04	0.39 (0.15-0.99)	.05
END	52 (38.8)	22 (35.5)	0.91 (0.62-1.36)	.66	0.91 (0.62-1.34)	.64

Effect sizes are shown as unadjusted risk ratio (RR) from the original population and adjusted RR (aRR) from the IPTW population. Adjusted for age, sex, pre-stroke mRS, hypertension, diabetes, atrial fibrillation, admission NIHSS, occlusion localisations (M1-segment, M2-segment of the middle cerebral artery, carotid-T, anterior cerebral artery, isolated extracranial carotid artery occlusion, tandem occlusion), intravenous thrombolysis and centre.

Abbreviations: CI = confidence interval; END = early neurological deterioration ≥ 4 NIHSS; ENI = early neurological improvement ≥ 4 NIHSS points; LVO = large vessel occlusion; mRS = modified Rankin Scale; sICH = symptomatic intracerebral haemorrhage; TICI = treatment in cerebral ischemia score.

In the subgroup analysis, no significant interaction was found between stent treatment and occlusion types (tandem occlusion, isolated extracranial carotid occlusion), stroke severity or intravenous thrombolysis/no-thrombolysis before thrombectomy (Table 3).

**Table 3:** Subgroup analysis of outcomes of endovascular therapy with and without emergent stenting.

	**mRS 0-2 at 3 months**				
	**Carotid stenting**	**No carotid stenting**	**RR**	***P*-value **	***P***_**interaction**_
Admission NIHSS ≤ 6 (*n* = 73)	19 (76)	38 (79.2)	0.87 (0.36-2.11)	.8	.64
Admission NIHSS > 6 (*n* = 328)	66 (64.1)	126 (56)	1.22 (0.91-1.65)	.2	.40
Isolated extracranial ICA occlusion (*n* = 113)	30 (66.7)	41 (60.3)	1.19 (0.72-1.98)	.5	.48
Tandem occlusion (*n* = 66)	14 (60.9)	27 (62.8)	0.95 (0.50-1.80)	1	.66
Intravenous thrombolysis (*n* = 186)	41 (71.9)	79 (61.2)	1.38 (0.86-2.21)	.2	.46
No intravenous thrombolysis (*n* = 223)	45 (60)	86 (58.1)	1.03 (0.85-1.24)	.9	.93

Effect sizes are shown as risk ratios (RR) and 95% confidence intervals (CIs). Interaction terms were tested in IPTW weighted regression models adjusted for age, sex, pre-stroke mRS, hypertension, diabetes, atrial fibrillation, admission NIHSS, occlusion localisations (M1-segment, M2-segment of the middle cerebral artery, carotid-T, anterior cerebral artery, isolated extracranial carotid artery occlusion, tandem occlusion), intravenous thrombolysis and centre.

Abbreviations: RR = risk ratio, mRS = modified Rankin Scale, NIHSS = National Institute of Health Stroke Scale, ICA = internal carotid artery.

## Discussion

This multicentre observational study evaluated functional outcomes and safety of thrombectomy with and without emergent stenting in patients with LVO stroke secondary to CAD. The main finding can be summarised as follows. Intraprocedural stenting led to higher odds of successful recanalisation and reduced 3-month mortality rates. Stenting appeared to be safe, with no increase in the sICH rates. However, it was not associated with more favourable functional outcomes, early neurologic improvement or early neurologic deterioration.

Although thrombectomy is an established therapy for acute stroke due to LVO regardless of aetiology, CAD may present unique technical challenges.^5^^,^^9^^,^^13-15^ Moreover, CADs remains an underrepresented aetiology in existing randomised trials.^16^^,^^24^^,^^25^ The current evidence supporting thrombectomy with carotid stenting primarily arises from patients with atherosclerotic occlusions.^10^^,^^11^^,^^16^^,^^17^ However, given some key differences between those etiologies, results may not be directly transferable.^25^ Indeed, thrombectomy with emergent carotid stenting in cases of tandem occlusions due to underlying dissection did not lead to improved functional outcomes as opposed to cases with an underlying atherosclerotic aetiology.^18^ Risk of false lumen stenting, an increased likelihood of intracranial embolisation and a potential necessity for the use of a second stent to cover long-dissected segments all contribute to the complexity of treating dissections.^13^^,^^19^^,^^25-27^ Previous studies have also associated cervical artery dissection with higher rates of stent occlusion.^19^^,^^27^

Nonetheless, observational data suggest that thrombectomy in CAD is safe and associated with higher recanalisation rates.^12^^,^^16^ A pooled analysis of the ETIS and TITAN registries and the recently published secondary analysis of the international Stroke Prevention in Cervical Artery Dissection (STOP-CAD) study are, to date, the only 2 studies addressing the role of emergent stenting in patients with LVO due to CAD.^10^^,^^11^ The population of both studies consisted of patients with tandem occlusions secondary to CADs. They found emergent stenting to be safe but without effects on favourable functional outcomes (mRS 0-2). However, rates of successful recanalisation (TICI 2b/3) were higher in the stenting group.^10^^,^^11^ These results are consistent with the findings of our study. Additionally, our study extends this observation to isolated extracranial carotid occlusions. Interestingly and in contrast to the 2 aforementioned studies our study found that CAS was associated with a reduction in mortality at 3-month follow up. A possible explanation for the observed difference in mortality rates is that patients who underwent stenting and who had higher rates of successful reperfusion were less prone to develop malignant cerebral infarction. Prior studies have demonstrated that successful recanalisation is associated with a reduced risk of malignant edema, which could partly account for the survival advantage observed in the stenting group.^28-30^

Limitations of our study need to be addressed. First, the observational design of this study leads to a potential bias by indication. Data on ASPECTS were not available, limiting the ability to assess the extent of baseline ischemic core. Similarly, data on symptomatic subarachnoid haemorrhage (sSAH) were not consistently collected and were therefore excluded from analysis, although sSAH represents a clinically important safety outcome. Furthermore, there were no standardised periprocedural antithrombotic regimens or stent surveillance protocols between the centres and data on these factors were not included in the analysis. This is of relevance given that CAD seems to be an independent risk factor for stent thrombosis which, in turn, is associated with poor functional outcome.^27^ Stent thrombosis could have potentially mitigated the clinical benefit of increased TICI 2b/3 recanalisation with carotid stenting. In analogy, missing information on distal periprocedural embolism, which seem to be more frequent with stenting^19^^,^^27^^,^^31^ may also have introduced a bias to the observation on functional outcome. Moreover, no data was available to differentiate hemodynamic versus embolic stroke mechanisms, although this may have influenced the choice of the treatment strategy. Assessment of functional outcomes and mortality was not blinded, which may have introduced an observer bias. Finally, approximately 25% of patients were lost to follow-up. While this may have reduced the reliability of outcome estimates, baseline characteristics did not differ between patients lost to follow-up and those with complete follow-up. Thus, our results need to be interpreted cautiously, with regard to the abovementioned limitations. On the other hand, the strengths of our study include large previously unreported multicentric observational cohort, rigorously collected clinical data and a setting closely reflecting real-life scenario.

## Conclusion

In patients with stroke due to anterior LVO associated with CAD, thrombectomy with intraprocedural stenting appeared safe, seemed associated with increased odds of successful recanalisation and reduced risk of mortality at 3 months. However, it was not associated with better functional outcome. Further clinical trials are warranted to clarify the optimal endovascular treatment strategy.

## Supplementary Material

aakaf004_Table_S1

aakaf004_Table_S2
