# Supplementary material for: Thrombectomy with and without emergent stenting in acute ischemic stroke due to carotid artery dissection
Source: Eur Stroke J. 2025 Dec 28;11(1):aakaf004. doi: 10.1093/esj/aakaf004 (PMC12866227; doi:10.1093/esj/aakaf004)
Supplement: aakaf004_Table_S1 [file aakaf004_Table_S1.docx]

Table S1. Comparison of patients with and without 3 months follow up.

|  | **Follow up= 409** | **No follow up= 107** | **p** |
| --- | --- | --- | --- |
| Age, years; mean (range, SD) | 53.8 (19-95, 11.8) | 53.9 (23-84, 12.7) | 0.8 |
| Sex, n (%) male | 312 (76.3) | 81 (75.7) | 0.9 |
| Admission NIHSS median (range, IQR) | 13 (0-42, 10) | 15 (0-42, 11) | 0.3 |
| Pre-stroke mRS 0-1, n (%) | 390 (95.4) | 100 (93.5) | 0.5 |
| Hypertension, n (%) | 174 (42.5) | 47 (43.9) | 0.8 |
| Diabetes mellitus, n (%) | 16 (3.9) | 8 (7.5) | 0.1 |
| Atrial fibrillation, n (%) | 16 (3.9) | 7 (6.5) | 0.2 |
| Vessel occlusion localization |  | | |
| M1, n (%) | 206 (50.4) | 51 (47.7) | 0.7 |
| M2, n (%) | 111 (27.1) | 25 (23.4) | 0.5 |
| Carotid-T, n (%) | 103 (25.2) | 18 (16.8) | 0.1 |
| Isolated extracranial ICA, n (%) | 119 (28.7) | 35 (34.7) | 0.3 |
| ACA, n (%) | 14 (3.4) | 0 (0) | 0.1 |
| Tandem, n (%) | 23 (21.5) | 66 (16.1) | 0.2 |
| Intravenous thrombolysis, n (%) | 186 (45.5) | 60 (56.1) | 0.1 |
| Door-to-needle time, min; median (range, IQR) | 39 (4-194, 40) | 38 (5-975, 53.3) | 0.8 |
| Door-to-groin time, min; median (range, IQR) | 75 (11-793, 65) | 81 (9-697, 62) | 0.7 |
| Duration of EVT, min; median (range, IQR) | 66 (9-541, 69) | 71.5 (9-488, 66.5) | 0.4 |

SD – standard deviation; NIHSS – National Institute of Health Stroke Scale; IQR – interquartile range, mRS – modified Rankin Scale, M1 and M2 – M1 and M2 segment of the middle cerebral artery, T- carotid terminus, ICA – internal carotid artery, ACA – anterior cerebral artery, EVT – endovascular therapy.
