# Supplementary material for: Thrombectomy with and without emergent stenting in acute ischemic stroke due to carotid artery dissection
Source: Eur Stroke J. 2025 Dec 28;11(1):aakaf004. doi: 10.1093/esj/aakaf004 (PMC12866227; doi:10.1093/esj/aakaf004)
Supplement: aakaf004_Table_S2 [file aakaf004_Table_S2.docx]

Table S2. Standardized mean differences (SMD) before and after IPTW.

|  | **Unweighted cohort** | | | **IPT weighted cohort** | | |
| --- | --- | --- | --- | --- | --- | --- |
|  | SMD | C.I. | | SMD | C.I. | |
| Age | 0.15 | -0.03 | 0.34 | 0.00 | -0.19 | 0.19 |
| Sex | 0.22 | -0.03 | 0.47 | 0.10 | -0.15 | 0.34 |
| Admission NIHSS | -0.10 | -0.29 | 0.08 | 0.02 | -0.17 | 0.20 |
| Pre-stroke mRS 0-1 | 0.15 | -0.34 | 0.64 | 0.09 | -0.40 | 0.59 |
| Hypertension | -0.80 | -1.01 | -0.60 | -0.01 | -0.22 | 0.19 |
| Diabetes mellitus | 0.23 | -0.23 | 0.69 | 0.06 | -0.60 | 0.48 |
| Atrial fibrillation | -0.05 | -0.55 | 0.45 | -0.10 | -0.64 | 0.43 |
| Vessel occlusion localization | | |  |  |  |  |
| M1 | -0.22 | -0.42 | -0.01 | 0.01 | -0.19 | 0.22 |
| M2 | 0.27 | 0.04 | 0.49 | -0.01 | -0.24 | 0.23 |
| Carotid-T | 0.10 | -0.13 | 0.34 | -0.02 | -0.26 | 0.23 |
| Isolated extracranial ICA | 0.16 | -0.05 | 0.38 | -0.02 | -0.25 | 0.21 |
| ACA | -0.10 | -0.75 | 0.55 | 0.02 | -0.58 | 0.62 |
| Tandem | 0.07 | -0.19 | 0.34 | 0.00 | -0.27 | 0.27 |
| Intravenous thrombolysis | -0.03 | -0.24 | 0.17 | 0.01 | -0.19 | 0.22 |
| Door-to-needle time | -0.00 | -0.26 | 0.24 | 0.09 | -0.16 | 0.35 |
| Door-to-groin time | 0.07 | -0.12 | 0.26 | 0.01 | -0.18 | 0.20 |
| Duration of EVT | 0.01 | -0.25 | 0.26 | 0.02 | -0.24 | 0.28 |

SMD – standardized mean differences; IPTW – Inverse probability of treatment weighting; aNIHSS – Admission National Institute of Health Stroke Scale; pmRS – Pre-stroke modified Rankin Scale, M1 and M2 – M1 and M2 segment of the middle cerebral artery, T- carotid terminus, ICA – internal carotid artery, ACA – anterior cerebral artery, EVT – endovascular therapy.
